# Alterations in the Coagulation System during Major Visceral Surgery in Children

**DOI:** 10.1155/2014/756809

**Published:** 2014-02-26

**Authors:** Hayarpi H. Kordjian, Mads Nybo, Niels Qvist

**Affiliations:** ^1^Department of Surgery, Odense University Hospital, 5000 Odense C, Denmark; ^2^Department of Orthopaedic Surgery, Vejle Hospital, Kabbeltoft 25, 7100 Vejle, Denmark; ^3^Department of Clinical Biochemistry and Pharmacology, Odense University Hospital, 5000 Odense C, Denmark

## Abstract

*Purpose*. The description of the alterations in the hemostatic system in children undergoing abdominal surgery is sparse. Enhanced clinical outcomes for previously untreatable conditions have led to an increased incidence of venous thromboembolic complications. Alterations in children's coagulation system during major abdominal operations compared to minor procedures were examined. *Methods*. Children (0–12 years) undergoing either laparotomy, thoracotomy, or minor surgery were included. Participants were divided into two groups: group 1 was open laparotomy including operations for solid abdominal tumours and thoracotomy, while group 2 was minor surgery. Activated partial thromboplastin time (aPTT), D-dimer, INR, and fibrinogen were measured. *Results*. Both groups had a shorter aPTT, higher INR, and lower fibrinogen concentrations after the operation, while D-dimer was unaltered. The changes were, however, discrete and probably not clinically significant. On day 3, all parameters except aPTT in group 1 (not measured in group 2) indicated a continuous coagulation activity. *Conclusion*. The tendency for coagulation activity altered based on the length and degree of surgery. A continuously altered activity was observed compatible with the reported increased risk of venous thromboembolism at day 3. However, before introducing thromboprophylaxis guidelines larger series of multicentre studies are needed.

## 1. Introduction

The knowledge on alterations in the coagulation system in children undergoing major surgery is sparse [1]. The assumption has so far been that it is comparable to adults, but the results have been limited due to practical and technical difficulties with blood collection from children [2]. Age-dependent aspects of the coagulation system have however previously been described [3], and Andrew et al. have presented reference values for a wide range of components in the hemostatic system in healthy premature infants and full-term newborns, respectively [4, 5]. Furthermore, reference values for maturation of the hemostatic system during childhood have been introduced [6], and it was concluded that, whilst functional, the hemostatic system in infants and children is in a process of development [4–6]. In general, plasma concentrations of most pro- and anticoagulant proteins are low during childhood compared with adult reference values [7], but these differences are thought mainly to be due to an age-related physiological difference and not an indication of any pathological process [1, 8].

Children regularly undergo major surgery including laparotomy and thoracotomy. It is generally accepted that children do not need prophylactic anticoagulant therapy because venous thromboembolic (VTE) complications are rarely seen in comparison to adults undergoing similar operations [6]. Also, the incidence of spontaneous VTE is very low in healthy children [4, 5]. However, due to advances in treatment and improved clinical results in children, who survive primary diseases with a previously fatal outcome, the incidence of VTE in paediatric patients has increased [9–11], most often caused by a combination of an underlying clinical condition and risk factors [12]. At particular risk are infants at three months of age and teenage girls [13, 14]. The main triggers are a central venous line (CVL), recent operation, immobilisation, cancer, chemotherapy, trauma, and various congenital disorders, while pathological conditions such as severe infections, sickle cell disease, and antiphospholipid syndrome are additional risk factors [15].

Limited knowledge of alterations in the coagulation system during surgery exists in this patient group. The aim was therefore to investigate the response of the hemostatic system in children undergoing major surgical procedures compared to minor.

## 2. Materials and Methods

### 2.1. Study Population

The inclusion criteria were elective or acute laparotomy, thoracotomy, or minor surgery in children aged 0 to 12 years admitted to Odense University Hospital in the period of February–October 2011. A total of 113 children were eligible for the study, and for 83 children the parents gave their consent. Exclusion criteria were a history of hemophilia, von Willebrand disease or Bernard-Soulier syndrome, newborns with an APGAR score below 7 at five minutes, and children with concomitant liver disease. The patients consisted of two groups: children undergoing open laparotomy or thoracotomy (group 1) and children undergoing minor surgery (group 2).  Table 1 describes the surgical procedures in each group. All operations were performed by two well-experienced paediatric surgeons. None of the children had excessive bleeding (10 mL/kg), and blood transfusions were not performed during the study.

### 2.2. Ethics

Informed consent was obtained from either the parents or custody holders of all children prior to participation. In case of shared custody both parents signed the consent form unless one of the custody holders, through a written proxy, had given allowance to the other part to make decisions on behalf of both. The study was conducted in accordance with the Helsinki Declaration and approved by the Local Ethics Committee (Project ID S-20100075).

### 2.3. Biochemical Analyses

The first set of blood samples was collected when intravenous cannulation for anaesthesia was performed. A second set was collected through the already inserted venous catheter immediately after completion of the operation. A third set of blood samples was taken three days after surgery from children who underwent a major operation and had a central venous line (CVL). During the study period the CVLs in all patients were in continuous use for therapeutic reasons, and thus none of the patients received heparin according to our hospital guidelines. Vacu-tubes (Terumo, type 9NC-363047) were used for blood collection. To avoid contamination with tissue materials in the blood samples and to optimise test results, 1 mL of blood was discarded prior to each sampling. All samples were centrifuged at 2500 rpm (for the D-dimer analysis at 4000 rpm) for 15 minutes at room temperature and then immediately separated into plasma or sera and stored at −80°C until analysis.

In order to elucidate alterations in coagulation and fibrinolysis, the following variables were selected: activated partial thromboplastin time (aPTT) covered the intrinsic and common coagulation pathways, INR (which includes coagulation factors II, VII, and X) covered the extrinsic coagulation pathway, fibrinogen represented the common coagulation pathway, and D-dimer was selected as an unspecific marker of activation of the coagulation system. They were all measured at a STA-R instrument (STAGO, Triolab A/S, Denmark) with dedicated reagents according to the manufacturer.

### 2.4. Statistical Analyses

As the study was purely descriptive, power calculations were not performed prior to the analyses. Preliminary analyses were performed to ensure that assumptions of normality, linearity, and homoscedasticity were not violated [16]. None of the four coagulation parameters were normally distributed, and nonparametric statistics were therefore applied. Wilcoxon paired test was performed for within-group comparison of two related samples, while between-group comparison of independent groups was tested by two-way ANOVA. The relationship between age and changes in the hemostatic parameters was investigated using Spearman's Rank Order Correlation Coefficient with two tailed *P* values. All *P* values < 0.05 were considered statistically significant. Analyses were performed using IBM SPSS Statistics 19.

## 3. Results

As shown in Table 1, a total of 48 patients were eligible in group 1, but nine declined participation and ten patients were excluded due to technical reasons (i.e., coagulated blood samples, insufficient amount of sample material, or difficulties in drawing blood). In group 2, 65 children were eligible, but 21 declined participation and 15 were subsequently excluded due to technical reasons as mentioned above. Baseline characteristics for the remaining patients are shown in Table 2. Not surprisingly, duration of surgery in group 1 was considerably longer than in group 2.

There were no significant correlations between age and the preoperative coagulation values (results not shown).

When coagulation parameters were compared in the two groups, preoperative values of D-dimer and aPTT values were slightly, but insignificantly, higher in group 1 patients, while INR and fibrinogen did not differ (Table 3).

**Figure 1:**
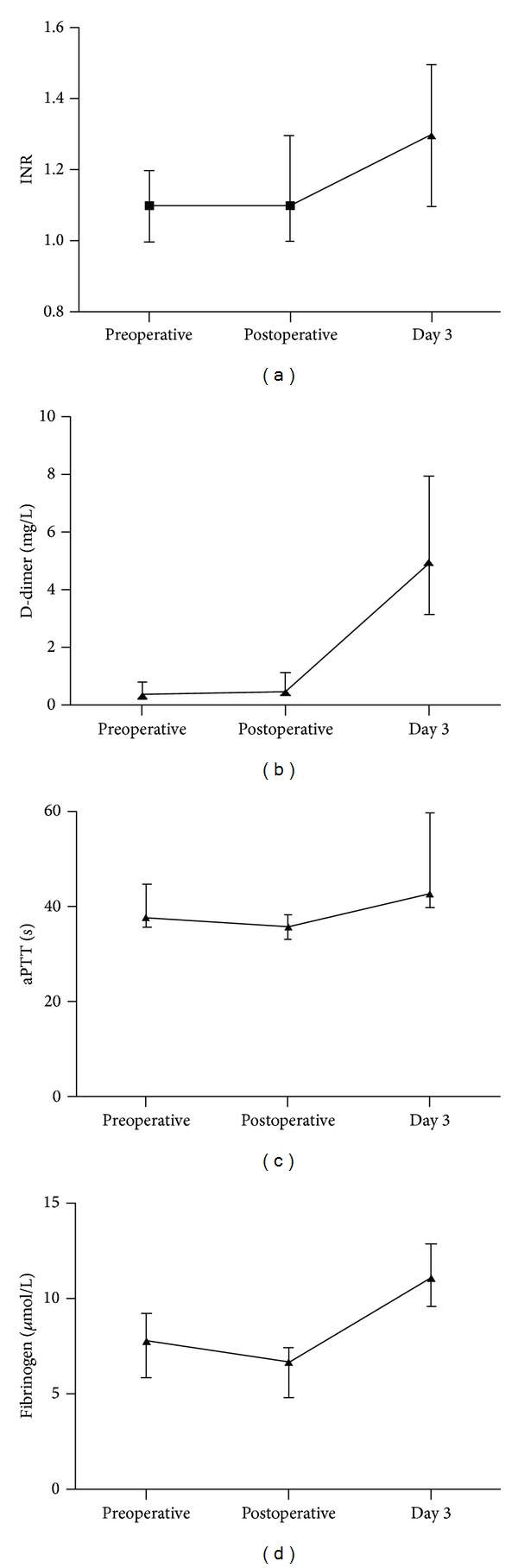
Pre- and postoperative values for coagulation parameters in the group undergoing major surgery. Values are medians and interquartile ranges (IQR). INR: international normalized ratio; aPTT: activated partial thromboplastin time.

In group 1, postoperative INR values increased significantly (*P* < 0.001) compared to preoperative values; however, the absolute increase was small and most likely not clinically significant. Fibrinogen and aPTT both decreased, while D-dimer was unaltered. Of interest, all four parameters were significantly altered on day 3 postoperatively indicating continuous coagulation system activation (Figure 1). In group 2, there was a small but significant increase from pre- to postoperative values in INR (*P* = 0.02) and a shorter aPTT (*P* = 0.03), while fibrinogen and D-dimer did not change.

## 4. Discussion

The natural history and risk factors for developing postoperative VTE in children are different from adults [17, 18], but the risk of VTE has only recently been thoroughly assessed in a paediatric population undergoing major surgery [19]. In order to develop evidence-based guidelines for the paediatric patients, improved understanding of changes in the hemostatic system during surgery is mandatory. We therefore conducted a study, which to our knowledge is the first with focus on alterations in the coagulation system in children during major and minor surgery.

Three findings emerged. Firstly, the two groups undergoing major or minor surgery did not differ preoperatively in coagulation status. Whether this is expected or not is difficult to state with such heterogeneous groups (Table 1), but patients admitted for major surgery would *per se* be considered more ill, and, therefore, a more vivid inflammatory response with its well-known connection to the coagulation system was expected. This was nevertheless not the case in our study, although a wider range of D-dimer in group 1 was observed with a maximum value of almost 15 mg/L in one patient.

Secondly, significantly different alterations in coagulation proteins between the two groups postoperatively were observed. A priori, major surgery would be expected to cause higher degree of coagulation activity and therefore also lead to a larger consumption of coagulation factors. The alterations after major surgery were significantly more pronounced, but the absolute value for the difference was small and was considered as not clinically significant. The difference may primarily be attributed to the significantly longer duration of surgery in this group (71 minutes versus 40 minutes in group 2). So again, the effect on the coagulation status does not seem to alter at a level that should contribute to an increased VTE risk in any of the groups.

Thirdly, when investigated at day 3 the coagulation system indeed did seem to have an increased activity in the group with major surgery. It would have been interesting to see whether the apparently favourable approach (minor surgery) was still favourable on day 3, but unfortunately we were due to ethical reasons not able to perform blood sampling on day 3 in this group. Nevertheless, we can conclude that major surgical procedures as performed in group 1 *did* trigger a long-lasting increased activation of the coagulation system, which could be compatible with the reported increased risk of thrombosis.

To our knowledge, there are currently no investigations of the influence of major abdominal surgery on the hemostatic status in children. However, there are few studies that illustrate hemostatic changes in cardiac, orthopaedic, and neurosurgery. The primary aim of these investigations was to study the VTE incidence in children.

- Manlhiot et al. found significantly increased VTE incidence in paediatric patients undergoing cardiac surgery due to a global inflammatory response triggered by the cardiopulmonary bypass procedure, which led to activation of the coagulation system [20].
- Kaabachi et al. studied VTE in teenagers who underwent surgery for idiopathic scoliosis [21] but did not find evidence for increased VTE risk secondary to spinal surgery. According to their study, children developing thrombosis had on average two predisposing/triggering prothrombotic factors (e.g., CVL).
- Goobie et al. used thromboelastography to evaluate the hemostatic changes in paediatric neurosurgical patients [22] and observed a hypercoagulate state in these patients although none of the patients developed VTE.
- Tabori et al. looked at the risk of clinically significant VTE in paediatric neurooncology patients [23] and found a remarkable difference in VTE incidence between adults and children with similar brain tumours. In adults, the use of high dose corticosteroids implicated an additional prothrombotic risk factor, whereas in children they suggested that other age-related factors such as vascular integrity and additional comorbid conditions have a strong influence on VTE risk.
- Perel et al. reported a case of portal vein thrombosis after splenectomy for hereditary stomatocytosis in a child [24], while Skarsgard et al. reported three cases of portal vein thrombosis after splenectomy for paediatric haematological diseases [25]. The latter found that only advanced age and splenic mass predicted thrombosis significantly.

None of these studies are directly comparable with our study, but the target population is somewhat similar as surgical trauma and stress inevitably affect the hemostatic system regardless of the type of operation.

Although our results indicate a continuous activation of the coagulation system postoperatively, we did not encounter any symptomatic events of thrombosis in these patients. This could indicate that thrombosis requires additional risk factors to develop as indicated by several of the mentioned studies and also known from patients with thrombophilia. However, it could also be due to the relatively low number of patients entering our study: the relatively small sample size might have caused a nonnormal distribution of data. Nevertheless, it is unfortunately a well-recognised problem with low participant numbers at this age, and we find that the data presented in our study are representative and sufficient enough to give a satisfying picture of the behaviour of the coagulation parameters investigated. Also, despite assistance of highly practiced anaesthetic teams well-trained in cannulation prior to surgical procedures, difficulties with blood collection were regularly encountered. However, the technical difficulties in blood sampling are compatible with reports from other research teams and are in general a critical issue that makes this research area challenging.

In conclusion, we were able to show alteration of the coagulation status related to the degree of surgery with a continuously increased coagulation activity at day 3 postoperatively compatible with the increased VTE risk reported. However, before introducing thromboprophylaxis guidelines in this cohort larger series of multicentre studies are needed to gather stronger evidence on this matter.

## Figures and Tables

**Table 1:** Surgical procedures in the two groups.

	Refused participation	Excluded	Included
Major procedures (group 1)			
Laparotomy			
Bowel resection and anastomosis	4	1	9
Colostomy	0	1	5
Diaphragmatic hernia repair	0	0	2
Duodenoduodenostomy	1	1	1
Explorative	1	1	2
Pancreatic resection	0	2	1
Splenectomy	0	0	1
Excision of neuroblastoma	0	0	1
Nephrectomy (Wilms' tumour)	0	0	2
Excision of benign cyst	1	1	4
Thoracotomy			
Esophageal atresia	2	3	1

Total	**9**	**10**	**29**

Minor procedures (group 2)			
Cryptorchidism	12	4	7
Hydrocele	2	1	2
Inguinal hernia	3	7	14
Umbilical hernia	4	3	6

Total	**21**	**15**	**29**

**Table 2:** Demographic characteristics of the two groups.

	Major procedures (*N* = 29)	Minor procedures (*N* = 29)
Boys/girls	20/9	27/2
Acute/elective	14/15	8/21
Median age (months)	14	21
Age range (months)	0–144	1–109
Weight (kg) (SD)	12.1 (7.7)	13.3 (6.3)
Duration of surgery (minutes)	71 (32)	40 (11)
Central venous line	17 (58.6%)	0

**Table 3:** Results of the different coagulation parameters in the two groups.

	Preoperative	Postoperative	*P* value
INR			
Group 1	1.1 (0.9–1.8)	1.1 (0.9–2.3)	<0.001
Group 2	1.1 (0.9–1.3)	1.1 (1.0–1.4)	0.02
*P* value	**0.18**	**0.04**	

aPTT (sec)			
Group 1	38 (27–200)	36 (27–64)	0.05
Group 2	37 (27–45)	35 (27–50)	0.03
*P* value	**0.05**	**0.3**	

Fibrinogen (*µ*mol/L)			
Group 1	7.8 (4.4–12.6)	6.7 (3.9–10.4)	<0.001
Group 2	8.1 (5.8–14.5)	7.8 (3.1–14.8)	0.07
*P* value	**0.29**	**0.009**	

D-dimer (mg/L)			
Group 1	0.39 (0.14–14.58)	0.46 (0.07–9.54)	0.8
Group 2	0.26 (0.13–0.55)	0.32 (0.15–20.00)	0.1
*P* value	**0.09**	**0.5**	
